# The Need for Studies to Evaluate the Reproducibility of the T-Wave Alternans (TWA), and the Rationale for a Correction Index of the TWA

**Published:** 2007-08-01

**Authors:** John E Madias

**Affiliations:** Mount Sinai School of Medicine of the New York University, New York, NY, and the Division of Cardiology, Elmhurst Hospital Center, Elmhurst, NY

**Keywords:** T-wave alternans, correction index

## Abstract

Sudden cardiac death (SCD) due to various cardiomyopathies is currently prevented by the implantation of an automated cardioverter/defibrillator (ICD). ICD impalntation in patients who are not survivors of SCD, or have not suffered potentially lethal ventricular arrhythmias, are based on the presence of cardiomyopathy with a reduced left ventricular ejection fraction. The bulk of patients who are considered suitable for an ICD implantation and receive such devices, do not experience device therapy shocks at follow-up ("false positives"), thus creating a climate of uncertainty among patients and physicians about the soundness of our current eligibility criteria for ICDs. In addition the cost of inappropriate ICDs is staggering, and the undue exposure of "false positive" patients to complications, and hardships is disconcerting. T-wave alternans (TWA) has emerged as a possible "risk detection of SCD" technology, but its reproducibility has not been tested. Peripheral edema (*extracardiac*) or other *cardiac* mechanisms, unrelated to the degree of SCD risk, alter the amplitude, and other attributes, of the T-waves. Since TWA may be T-wave amplitude-, or other T-wave attributes-dependent (this is still a speculation), a need may be emerging for its correction by the T-wave amplitude (TWA index); such an index may enhance the reproducibility, and evaluate the true sensitivity, specificity and predictive accuracy of the TWA in detecting future victims of SCD.

Sudden cardiac death (SCD), which is due primarily to ventricular tachycardia and fibrillation, accounts for ~450,000 deaths annually in the USA [1] The great majority of the victims have ischemic cardiomyopathy consequent to a large myocardial infarct and/or ischemic dilated cardiomyopathy, or non-ischemic cardiomyopathy, due to a variety of other pathlophysiological etiologies [2]. Many of these patients have clinical congestive heart failure (CHF), and experience dyspnea, fatigue, initially with exertion and subsequently at rest, and SCD. Also such patients have systolic dysfunction, as assessed by a reduced left ventricular ejection fraction (EF), assessed by different imaging modalities. An estimated 4.9 million people in the USA have CHF, with ~550,000 cases reported annually [2]. Although the implementation of the implantable cardioverter/defibrillator (ICD) has been found extremely effective in aborting SCD and thus saving lives [3-5], these devices are implanted to thousands of patients with low EF and CHF, who over the course of follow-up do not receive life saving shocks due to the overall low incidence of SCD in these populations [6].  Indications for ICD implantation are resuscitated SCD, sustained ventricular tachycardia, CHF, and low EF [5]. Prolonged QRS complex duration has been considered a pathology index for follow-up and prognosis in patients with CHF and in the evaluation for ICD implantation [7]. This index however is under scrutiny, and of late has been considered less reliable than the EF, in fathoming the suitability of patients for ICD implantation [8]. There is a compelling need for employment of additional parameters, to the clinical picture and EF, in the selection of patients with CHF who have not experienced lethal arrhythmias (the bulk of such patients), but who nevertheless may need ICD for prevention of SCD; also the majority of patients with this clinical profile after having an ICD implanted, do not receive shock therapies at follow-up, suggesting that probably the ICD implantation was not indicated after all, or generating ambivalence to the physicians and patients alike, as to the soundness of our current criteria for employing these invasive devices.

## T-wave alternans

Recently T-wave alternans (TWA), which reflects in its simplest form a beat-to-beat variation in the T wave shape, timing, and amplitude, or cardiac repolarization in general [9], has emerged as such plausible parameter. On March 21, 2006, the Centers for Medicare and Medicaid Services (CMS) issued a decision memo stating that the "Microvolt T-Wave Alternans diagnostic testing is covered for the evaluation of patients at risk for sudden cardiac death"; this coverage is provided only "when the analytic spectral method is used" for the calculation of TWA [10] (vide infra). TWA varies in its presentation from a macroscopically (≥50 μV), easily visually appreciated form (described almost 100 years ago) to a microscopically occurring disturbance, measuring a few μV in amplitude, which requires special algorithms for its detection. The beat-to beat variation may range from alternating change in amplitude of the T-waves to alternating T-waves of opposite polarity. TWA is calculated with a variety of methods, taking into consideration various attributes of cardiac repolarization. Two methods have emerged and are dominant currently, one employing spectral analysis using the fast Fourier transformation [11], and another employing a time domain analysis, using the "modified moving average" approach [12] with the former implemented in a commercially available device (Cambridge Heart, Bedford, MA) [13]. The patient is tested either invasively in the electrophysiology laboratory, where cardiac pacing is being used for its elicitation [14,15], or noninvasively (the currently favored approach) in the context of exercise testing (bicycle ergometry, or treadmill) [16], with the purpose of modestly increasing the heart rate, or by implementing ambulatory (Holter) ECG monitoring [12]. Additionally if exercise is not thought to be feasible in a patient, pharmacological means (e.g., dobutamine) for increasing the heart rate may be used in the implementation of TWA testing. A rise in heart rate is required for emergence of TWA, since even patients with severe CHF do not show TWA at rest, but only very rarely. Since TWA with rapid heart rates may not be pathological, and may even be elicited in normal individuals ("false positive" response), there is a critical rise in the heart rate (usually ≤110beats/min), which is recommended for the assessment of clinically meaningful detection of TWA [13-17]. Also the magnitude of TWA in μV is dependent on the heart rate and thus pacing-, or exercise-induced heart rate rise within a narrow range elicits progressively larger responses of TWA values in μV [18]. The last attests to the dependence of the magnitude of TWA on the heart rate rise. TWA measurement, using the above 2 methods [11,12]. elicits values of different order of magnitude, with the spectral method reporting much lower values, and mainly in a qualitative form with an abnormal cutoff point of ≥ 1.9 μV. Nevertheless reporting data in quantitative form has been increasingly encouraged and felt to be advantageous [18,19]. The phenomenon of TWA may assume complex forms [9], but the clinically evaluated, and the commonest form, is that of the alternating beat-to-beat variation in the T-wave. Recently the elicitation of TWA employing simultaneous ventricular and atrial stimulation in the context of an electrophysiologic procedure has been implemented in patients who have suffered an acute myocardial infarction, and has been found to have superior sensitivity and specificity, and a lower rate of indeterminate tests (vide infra), over the procedure using atrial stimulation, or the noninvasive version based on exercise testing [20]. Most recently a comparison of TWA assessment using atrial versus ventricular pacing, showed the latter to be superior [21]. Thus the way to enhance sensitivity and specificity and to minimize the rate of indeterminate tests appears to be an invasive-based procedure. Of note is that while TWA measurements/calculations/mathematical data treatment, when based on exercise testing or right atrial pacing, are carried out on the repolarization signals of intrinsic ventricular beats, when based on ventricular pacing, the repolarization signals consequent to an artificially paced ventricular beats are dealt with.

## Reproducibility of T-wave alternans

The reproducibility of a diagnostic test is of paramount importance for reliance on it, for decision-making. Thus it is expected that back-to-back performance of a test should lead to reasonably similar values; also tests performed hours, days, weeks, or even years apart should generate reasonably close values, providing that the clinical status of the tested patient is about the same during the 2 testing incidences. The EF e.g., the main laboratory parameter along with clinical assessment currently employed in the selection of patients for ICD implantation, is expected to be stable when tested repeatedly with the same method, and provided that no worsening or improvement of the patient has been documented in the intervening time period. In fact when EF values are significantly different on repeat testing, an investigation is launched to detect the reasons for such discrepancy. The same reproducibility, relied upon in different modalities, should be expected from TWA. Long-term reproducibility of TWA has not been evaluated. As of July 7, 2007, 387 titles were listed in Pub Med in response to the inquiry phrased as "T-wave alternans; none of these entries actually deals even tangentially with the problem of reproducibility of the method. By this I mean comparing data from 2 TWA tests (of any type), done at 2 different time points (days, months, or years apart), in a cohort of clinically stable patients, ensuring that measurements were made at identical heart rates. Only a few studies measuring short-term reproducibility have been published [14-17], and they were based on back-to-back testing or tests performed a few hours apart, or involved use of a drug during the repeat TWA testing (not real reproducibility studies) [14,15]. Studies on short-term and long-term reproducibility are needed, if  TWA testing is to become a clinical tool for evaluation of patients with CHF for ICD implantation. It needs to be demonstrated that TWA is present or absent, or in the former situation of a certain stable value of magnitude, when a patient with CHF is at the same state of clinical compensation, as assessed by symptoms, signs of disease, and routine laboratory testing.

## Magnitude of T-wave alternans

An important issue of concern, among others, is the current consideration that a patient is "positive" when a TWA ≥1.9 μV is found on testing [9], *which appears counterintuitive to what is expected in biological systems*. An *absolute* "cutoff point" or threshold above which pathology is ascribed must be suspect; accordingly the increasing magnitude of TWA with increasing heart rates, the association with more severe pathology when TWA is found at rest or with slow heart rates, and the recently reported notion that "size matters after all" [19,22], suggests that TWA should become a quantitative assessment. However the issue of the variation of the TWA in response to the increasing heart rate during exercise testing is not a problem in implementing TWA testing to be solved, since exercise is confined to levels that increase the heart rates to ~110 beats /min [9]. This also applies to the invasive assessment of TWA by pacing [14,15,20,21]. Nevertheless pacing the patient incrementally, or exercising so that data on TWA are provided at heart rates of 90, 100, 110, and 120 beats per minute may be important when comparing repeat TWA tests of a single individual, or a cohort of patients. TWA is also affected by several other mechanisms, including autonomic nervous system tone, and various drugs [23]. Adrenergic stimulation elicits or enhances TWA [24], and beta-blockers lead to attenuation of the TWA [14,15]. However it has been suggested that for patients with CHF taking beta-blockers, there is no need to stop these essential drugs prior to testing the patients for TWA [9,14]. Indeed intuitively it appears appropriate to implement TWA testing while the patients are on their "routines", in terms of their maintenance drugs, since in such a setting also experience their risk of SCD. Finally a disconcerting matter is that of the "indeterminancy" of TWA tests, and the notion that there is scientific justification for considering such tests as "positive" [25], which makes some uneasy, about such trend [26].

## "T-wave amplitude dependence" of the T-wave alternans

Although more problems may be encountered, as the TWA testing is being implemented in practice and research, the balance of the present exposition focuses on the change in the T-wave amplitude as confounding factor in the implementation of this modality. These issue needs to be addressed presently for optimization of the application of the TWA testing [27,28]. Finally the reproducible placement of the limb, and especially precordial, leads in repeat TWA testing cannot be overemphasized; vast experience in the exercise stress laboratory has shown that deviations from the standard positioning of recording electrodes (unfortunately a very common encounter) can cause major changes in the amplitude, shape and duration of T-waves, and thus possibly affect the magnitude of TWA (vide infra). Such variations in the positioning of the precordial lead electrodes on the chestwall are particularly important, since they are the most useful (e.g., V2-V4), or often preferred, for TWA analysis [19].

## Peripheral edema and T-wave amplitude

Changes in the amplitude of T-waves may be traced to electrophysiological causes (*real*), or extracardiac mechanisms (*apparent*). A supportive argument for the latter is the recent demonstration that peripheral edema (frequently present in patients with CHF) results in reversible attenuations of the amplitude of the P-waves, QRS complexes and T-waves [29-34], with corresponding shortening of QRS complexes and QTc intervals [35,36]. All these changes are not electrophysiologically mediated, but are the result of the increased fluid content of the electrically "passive" volume conductor enveloping the heart due to fluid volume overload. It should be envisaged that TWA happens at the action potential, extracellular, and epicardial levels, but electrical currents of its signature are transmitted to the recording sites at the body surface, and in the process undergo transfer alterations due to the intervening inhomogeneous collection of organs and tissues [37-39]. Why should the magnitude of TWA be "immune" to such influences, if all other amplitudes and intervals are influenced by the changes in the extra-cardiac milieu, and if TWA is dependent on the T-wave amplitude? (vide infra). If one considers for a moment that not only the presence, but also the magnitude, of TWA is of clinical significance the reported attenuation of the ECG voltage [29-34] must have some effect on the TWA value. T-waves are also subjected to reduction in amplitude in edematous states [34] including CHF, and such amplitude attenuations possibly may affect the values of TWA. It should be emphasized here that such fluid overload conditions are frequent in patients with CHF, even if they are not clearly clinically apparent; the patient with CHF may have accumulated several liters of interstitial fluid before peripheral edema becomes clinically apparent [40]. Theoretically if a patient with CHF has e.g., TWA of 5.0 μV magnitude in state "A", based on measured T-wave amplitude of 200 μV in beat "a" in e.g., lead V3, and 195 μV in beat "b", a supervening edematous state "B", which has imparted a 50% reduction of the ECG voltage, from the onset of the P-wave to the offset of the T-wave, will result in T-waves of 100 μV in beat "a", 97.5 μV in beat "b", and a calculated TWA of 2.5 μV. Even if one considers that the absolute value of ≥1.9 μV in a patient suffices to characterize the testing for TWA as "positive", the emergence of the above described *extracardiac* mechanism could lead to a TWA value of <1.9 μV, and a "negative" test. In a more complex model, where both changes in the body volume conductor, and *cardiac* electrophysiological alterations are expected, it will be problematic to separate actual (*electrophysiologically mediated*), from apparent (*extracardiac*) influences on the magnitude of TWA. Moreover changes in the amplitude of T-waves occur occasionally in clinically stable patients without an apparent reason; such changes in the T-wave amplitude may lead to calculated TWA of different magnitude (as per argument above) when quantitation is used, or render a different qualitative characterization ("positive", "negative", or "indeterminate"). In addition to *apparent* (in the setting of CHF), *real* (electrophysiologically mediated) changes in the amplitude of T-waves should not be always considered as reflecting variations in the SCD risk, but may be due to a large variety of influences known to change the T-wave amplitude; nevertheless such T-wave amplitude changes might also possibly engender changes in the TWA magnitude, as argued above.

## A need for a T-wave alternans INDEX

Based on the above, what has been proposed is an index of TWA (TWAI) [41], which corrects, adjusts, or normalizes, for the specific amplitude of the T-waves at the time point of a particular assessment of TWA. The correction is applied to the output of any conceivable algorithm measuring/calculating the TWA, and includes the spectral measurement [13], the time-domain analysis using the "modified moving average" [9,12] or any future mathematical treatment of the *T-wave/overall repolarization* data. Such output values in μV are placed in the numerator of a fraction, with the amplitude of the corresponding T-wave in μV placed in the denominator. By the "T-wave" herein it is implied the taller T-waves of the 2, in the "ababab" sequence encountered in the phenomenon of TWA. Also different data, instead of the T-wave amplitude, can be substituted in both the numerator and the denominator and depending on the algorithm implemented in the measurement/calculation of the "TWA". *Thus if other than amplitude information pertaining to parts of the T-wave, or ST-segment, or combinations are used for the TWA assessment (numerator), similar information derived from the taller T-wave will be substituted in the denominator*.  This correction of TWA data can be applied to the T-waves of individual leads 1, aVF, and V1(V2), or leads V2-V4,  or the orthogonal Frank leads, or the vector magnitude lead.  In the last situation the denominator will comprise the square root of the sums of the squared values of the amplitudes of the T-waves from the 3 contributing leads [41]. The TWAI, although a theoretical construct, has plausible physiological underpinnings. Whether it has any incremental value would be decided upon by its implementation in research and practice.
